# Perception of *Deqi *by Chinese and American acupuncturists: a pilot survey

**DOI:** 10.1186/1749-8546-6-2

**Published:** 2011-01-20

**Authors:** Kathleen KKS Hui, Tara N Sporko, Mark G Vangel, Ming Li, Jiliang Fang, Lixing Lao

**Affiliations:** 1 Department of Radiology, Massachusetts General Hospital & Harvard Medical School, Charlestown, Massachusetts, 02129, USA; 2 Department of Radiology, Guang AnMen Hospital, China Academy of Chinese Medical Sciences, Beijing, 100053, China; 3 The Center for Integrative Medicine, University of Maryland School of Medicine, Maryland, 21207, USA

## Abstract

**Background:**

In acupuncture, *deqi *is the sensory experience related to clinical efficacy. As the first study taking into account cultural differences on *deqi *sensation, this pilot survey aims to corroborate the acupuncturists' general experience in clinical practice with functional magnetic resonance imaging (fMRI) findings.

**Methods:**

Questionnaires were distributed to acupuncturists of TCM (traditional Chinese medicine)hospitals and acupuncturists attending workshops and seminars in the United States and China. Questions covered clinical significance of *deqi*, patient attitude and the nature of some pain-related sensations elicited by manual needling.

**Results:**

47 out of a total of 86 acupuncturists agreed that dull pain was *deqi *and over half regarded it beneficial, while sharp pain was non-*deqi *and harmful instead. The patients' attitude toward *deqi *sensation showed a difference between US and China. There was no other dimension showing a difference.

**Conclusion:**

Results of this pilot survey indicate that the acupuncturists' perception is consistent with our previous fMRI findings. Results showed almost complete agreement that dull pain is considered *deqi *and beneficial to treatment, while sharp pain is not *deqi *and harmful. Particularly, dull pain was *deqi *and was beneficial to treatment whereas sharp pain was not. Patients in China liked the *deqi *experience whereas those in the US did not.

## Background

Acupuncture stimulation elicits a sensory response termed *deqi *which literally means "the arrival of vital energy" in traditional Chinese medicine *(*TCM). Multiple unique sensations experienced by the patient around the site of needle manipulation are often described as *suan *(aching or soreness)*, ma *(numbness or tingling)*, zhang *(fullness/distention or pressure) and *zhong *(heaviness) [1]. While pain is also experienced occasionally, the type of pain has not been well characterized. The increased resistance of the needle is felt by the acupuncturist (needle grasping) as tense, tight and full like "*a fish biting onto the bait*." as described in the literature [1-3]. Needle grasping is believed to be related to clinical efficacy [1-4] although little data are available [5-7]. The acupuncturist's skills, competence and understanding of the TCM theory also play an important role in the therapeutic outcome [8].

Randomized, placebo-controlled clinical trials of acupuncture evaluate its efficacy by separating the specific effects from the non-specific ones [8]. While a number of clinical trials failed to find *verum *acupuncture more effective than sham acupuncture in migraine [9,10] low back pain [11,12] and knee osteoarthritis [13,14], both *verum *and sham acupuncture were more beneficial than the waiting control. However, many of the studies included in the analysis did not satisfy the criteria for dosage adequacy required for optimal clinical efficacy [15] and that sham is not necessarily inert [16,17]. The intensity of the psychophysical and neurological response of *deqi *is now proposed to serve as a basis for dosage measurement [15,18], calling for a better understanding of the qualitative and quantitative characterization of the *deqi *sensation.

Most studies described the qualitative characteristics of sensations unique to acupuncture based on interviews of patients and expert acupuncturists [5,19]. Recently, several groups created quantitative sensation scales and *deqi indices *based on pain questionnaires distributed to acupuncture patients [5,20-25]. Classical sensations such as aching, soreness, numbness, fullness and heaviness were included. Sharp pain is not regarded as a beneficial *deqi *sensation by most acupuncturists. Most reports failed to separate it from dull pain in the categorization of *deqi *response; only a few reports with quantitative measures distinguished sharp pain from *deqi *[15,20,22]. We attempted to separate the acupuncture sensations into two major categories, namely sensations that do not hurt (aching, soreness and dull pain) as *deqi *and sensations that do hurt (sharp pain) as noxious stimulation [26,27]. Our findings showed that the *deqi *response was elicited in 71% acupuncture versus 24% tactile and that the frequency and intensity of sensations were significantly greater than tactile. Dull pain was significantly different from the tactile group among the gamut of sensations comprising *deqi*. Importantly, we have consistently observed distinct patterns of limbic network hemodynamic response in the brain, namely deactivation in *deqi *and activation in sharp pain. Our findings are in fair agreement with Macpherson's report [22] and the Southampton group [25]. Taken together, these psychophysical and hemodynamic response patterns may provide a qualitative and quantitative measure of the patient's response to manual needle manipulation [26-28].

This pilot survey aims to provide a picture by focusing on the items that are seldom described such as the subject's throbbing and dull pain and the acupuncturist's grasping sensation, serving as a foundation for a better understanding of acupuncture effects.

## Methods

### Survey design

The questionnaire was created collaboratively by the clinical acupuncturists and research staff at the Athinoula Martinos Center of Biomedical Imaging (Charlestown, MA, United States). Instead of the traditional sensations such as soreness, numbness and heaviness, we focused on sensations that are often overlooked (*ie *dull pain, sharp pain and throbbing).

Paper questionnaires were answered by licensed Chinese medicine practitioners on a self-reported and voluntary basis. Participants were asked whether they considered a specific type of pain-related sensation elicited from manual needling was *deqi *and whether it was beneficial or harmful to the patient. The questions were designed in a multiple-choice format and tailored to specifically address the nature of acupuncture sensations and its clinical significance based on the experience of the patient and the acupuncturist. Besides definitive answers, the participant was allowed to select "unsure" and allocated space for comments.

### Survey administration

Fifty questionnaires were distributed to licensed acupuncturists attending workshop seminars directed by one of the co-authors (LL): 30 in Chicago (USA) and 20 in Shanghai (China). Questionnaires were also distributed to acupuncturists in Chinese medicine teaching institutions: 20 to the School of Traditional Chinese Medicine in San Francisco (United States) and 6 to Guang'anmen Hospital of the China Academy of Traditional Chinese Medicine in Beijing (China). Another ten questionnaires were distributed via email to the Beijing University of Chinese Medicine (China). Questionnaires were bilingual in English and Chinese. Survey participation was on a voluntary basis and did not involve patients; no consent form was needed. The acupuncturists' names were kept confidential. The distributors did not participate in the design of the questionnaires but in data analysis.

### Statistical methods

A test of two proportions using two-tailed Fisher's Exact test was used to compare the sample proportions from the two countries using the conventional criterion for statistical significance, alpha = 0.05. Analyses were performed using Minitab v15 (Minitab^® ^Statistical Software, USA).

## Results

### Participation rate

Of the 86 questionnaires distributed, 47 were returned, representing a 55% response rate. Of the 47 respondents, 26 were from the US and 21 from China. Four of the returned questionnaires from the US were excluded from analysis because the respondents indicated that the questions were unclear to them. The total of 43 returned questionnaires pooled from both countries for the final analysis represented 50% of the 86 questionnaires distributed and 92% of the 47 respondents. All the respondents in this study identified themselves in the survey as those practicing acupuncture of traditional Chinese style.

### Participants

The training experience for participants pooled from the two countries averaged 6.4 years (range 2-18). Experience in practice averaged 12 years, (range 1-32). A larger range was observed for acupuncturists in China in years of practice (1-32 years) than those in the US (1.5-17 years).

### Relative importance in clinical efficacy: subject's vs acupuncturist's sensations

When asked whether the patient's or the acupuncturist's *deqi *experience is more important for clinical efficacy (Table 1), an overwhelming majority (77%) of participants selected "both the acupuncturist's *deqi *experience and the subject's *deqi *experience are important" without favoring one or the other. A small number, 12%, favored the patient's experience and 5% favored the acupuncturist's.

**Table 1:** Response of acupuncturists to by country

		US		China		Pooled		*z*	*P*
Question	Response	*n*	%	*n*	%	*n*	%		
*What deqi sensory*	Both the patient and acupuncturist	16	72.7	17	81	33	76.7		
*experience is more*	Patient	3	13.6	2	9.5	5	11.6		
*improtant for clinical*	Acupuncturist	1	4.6	1	4.8	2	4.7		
*efficacy?*	Blank	2	9.0	1	4.8	3	7.0		

*What is the relationship *	Occur together	7	53.8	5	31.3	12	41.4		
*between needle grasping and*	Not related	0	0.0	1	6.2	1	3.4		
*patient deqi experience?*	Sometimes occur together	2	15.4	7	43.8	9	31.0		
	Unsure	4	31.0	2	12.5	6	21.0		
	Blank	0	0.0	1	4.8	1	3.4		

*Do patients *	Yes	2	9.1	15	71.4	17	40.0	5.37^***^	0.000
*like the sensations of deqi?*	No	8	36.4	0	0.0	8	18.6	3.55^**^	0.004
	Neutral	6	27.3	6	19.1	12	23.2		
	Not felt	0	0.0	1	4.8	1	2.3		
	Other	2	9.1	1	4.8	3	7.0		
	Blank	4	18.2	0	0.0	4	9.3		

*Does a correlation exist *	Positive	13	65.0	17	81.0	30	73.2		
*between deqi and clinical*	Negative	1	5.0	0	0.0	1	2.4		
*efficacy?*	Unsure	1	5.0	3	14.3	4	9.8		
	Other	0	0.0	1	4.8	1	2.4		
	Blank	5	25.0	0	0.0	5	12.2		

Significant difference was observed between cultures in patient attitude: Test of two proportions between China and US using Fischer's exact *P*-values showed patients in China liked the sensations while those in the US did not (***P < 0.001; **P < 0.01).

### Relationship between acupuncturist's needle grasping sensation and patient's deqi response

Participants were asked if there was a relationship between the needle grasping sensation felt by the acupuncturist and the patient's *deqi *experience (Table 1). Most respondents indicated that the acupuncturist's needle grasping and the patient's *deqi *experience occurred concurrently (41%) or that sometimes they occurred concurrently (31%). Almost a quarter of respondents (24%) was unsure or not providing an answer.

### Patient's attitude towards deqi sensations

Participants were questioned about their patient's attitudes towards *deqi *sensations (Table 1). A total of 40% of participants reported that patients liked the *deqi *sensations whereas 19% reported "dislike" and rest neutral. There was an interesting difference between the two countries regarding the *deqi *sensation. Of the 17 respondents who indicated that the patients liked the sensations, only 2 were from the US and all the rest from China (*P *< 0.0001). In contrast, of all returned questionnaires that indicated "dislike" (19%), all were from the US and none from China (*P *= 0.0020).

### Correlation between deqi response and efficacy

Participants were asked whether there was a correlation between *deqi *and clinical efficacy in their acupuncture practice (Table 1). A total of 30 respondents (73%) indicated a positive correlation; no cultural differences among the respondents.

### Types of pain and deqi

Participants were asked to indicate "*deqi*" or "not *deqi*" for sensations often associated with pain, such as throbbing, dull pain and sharp pain and whether these sensations were beneficial or harmful. Of all participants, 72% agreed that dull pain was characteristic of *deqi *and beneficial to clinical efficacy (53%). Only 2-3% indicated dull pain was "not *deqi" *and harmful. About 20% of returned questionnaires did not have the answer for the question.

Very few participants considered sharp pain to be *deqi *or beneficial (7% and 14% respectively) and 50% classified it as "not *deqi" *and 42% believed it was harmful.

Respondents were less certain about the nature of throbbing and its relation to efficacy than they were about dull pain or sharp pain. Responses were almost equally divided between "*deqi*" (28%) and "not *deqi*" (21%). While over two-thirds answered "unsure" or did not provide an answer, 14% considered throbbing as "beneficial" and 21% "harmful".

In summary (Table 2), the majority of respondents considered dull pain to be *deqi *(91%), beneficial and clinically efficacious (96%). Conversely, 88% of respondents considered sharp pain to be "not *deqi*" and 75% considered sharp pain harmful and non-efficacious. In respect of throbbing, while respondents who opted for no answer or 'unsure' were the majority, no significant differences were observed between those favoring *deqi *and beneficial or non-*deqi *and harmful.

**Table 2:** Survey responses by country in respect of pain characteristics

Summary of responses		Dull pain		Throbbing		Sharp pain	
		*n*	%pooled	*n*	%pooled	*n*	%pooled
*Deqi *vs. Not *deqi*							
	*Deqi*	31	91.2	12	57.1	3	12.0
	Not *deqi*	3	8.8	9	42.9	22	88.0
Beneficial vs. Harmful							
	Beneficial	23	95.8	6	40.0	6	25.0
	Harmful	1	4.2	9	60.0	18	75.0

## Discussion

### Limitations

The sample size of this study was small and the scope of this survey is narrow. The exclusion of the timing of *deqi *assessment and sensations is also a limitation of this study. We consider a participant's reliance on memory in respect of sensation timing may result inaccuracy in a survey; therefore, we advocate a detailed exploration of dosage and patient attitude in an apposite clinical lab setting.

### Relation between deqi and clinical efficacy

Most participants (77%) in both countries believed there was a positive correlation between *deqi *and clinical efficacy. Our fMRI studies found a positive correlation between a subject's psychophysical and hemodynamic response: *ie *strong *deqi *sensations induced strong deactivation of the limbic system [27,28]. Such correlation provides strong evidence, albeit indirect, that neurophysiological effects of acupuncture *deqi *are mediated via specific brain networks.

Most participants indicated that both the patient's *deqi *and the acupuncturists' grasping sensation were important. However, this pilot survey found that a small percentage of participants disagreed with the majority, with 12% favoring the patient's experience and 5% favoring the acupuncturist's sensory experience.

### Dull pain, sharp pain and throbbing

Of the three types of pain related sensations, dull pain was generally considered to be *deqi *and beneficial while sharp pain was considered to be unrelated and noxious instead. In regard to throbbing, about half of the respondents were uncertain; the rest were almost equally divided between the two categories. Dull pain is seldom described in acupuncture literature. Park and Lee mentioned a 'dull' sensation but did not specify whether it was pain-related [23]. We first called attention to dull pain as a characteristic component of *deqi *and noted that it often occurred before or independent of sharp pain instead of occurring after sharp pain as described in the literature [26,27]. This pilot survey found that most respondents indicated dull pain as *deqi *and beneficial and sharp pain as noxious and harmful. These sensations and their relationship to *deqi *are often overlooked/neglected sensations. We regard their importance as we encounter them in our studies. With fMRI monitoring, we demonstrated that dull pain deactivated the limbic system and that sharp pain activated it [27,28]. Our findings are in agreement with MacPherson's report [22].

The survey results on throbbing were less definitive than dull pain or sharp pain; most participants were unsure of its nature and the rest were equally divided between *deqi *and not *deqi *as well as between beneficial and harmful. There is little information on throbbing in the literature. Occasional reports suggest that it occurs more often in electro- than in manual acupuncture; it's association with neuronal activity is unclear [5,23]. Our fMRI studies found that this sensation could be associated with either predominant deactivation or activation of the limbic system and pain matrix; throbbing may represent an intermediate stage in the excitation of the fine pain conducting fibers; a progressive increase in the intensity of mechanical stimulation may cause transition from the more innocuous dull pain to throbbing and finally to overt noxious sharp pain.

The significant difference between the two countries in respect of the patients' attitudes towards *deqi *sensation may have clinical implications. Most respondents in China reported that patients liked the *deqi *sensations while those from the US disliked them. Interestingly, this was the only response that showed significant difference between the two countries. A difference in patients' attitudes may affect how acupuncturists adjust their techniques and intensity of stimulationto the sentiments of his patients. This raises an important question as to how TCM practices are translated and practiced across cultures and uncovers confounds in meta-analysis of clinical efficacy studies. It is possible that acupuncturists in the US adopt less intensive and shorter durations of stimulations out of consideration for the patients' attitudes towards the sensory experience, resulting in lower dosage levels and lower therapeutic effects. Further investigation with large clinical sample sizes is warranted.

## Conclusion

Results of this pilot survey indicate that the acupuncturists' perception is consistent with our previous fMRI findings. This survey contributes valuable information regarding the opinions of practicing acupuncturists on the nature of *deqi *in U.S. and China, to support our fMRI findings. Particularly, dull pain was *deqi *and was beneficial to treatment whereas sharp pain was not. Patients in China liked the *deqi *experience whereas those in the U.S. did not.

## Abbreviations

fMRI: Functional Magnetic Imaging; TCM: Traditional Chinese Medicine; US: United States

## Competing interests

The authors declare that they have no competing interests.

## Authors' contributions

KKSH and LL conceived the study design and interpreted the data. KKSH wrote the manuscript. LL finalized the manuscript. TS performed data analysis and helped write the manuscript. ML performed the acupuncture in our acupuncture database in our previous studies. MGV conducted and supervised statistical analysis. JF participated in the design of the questionnaire. All authors have read and approved the final version of the manuscript.
